# The ALSFRS as an outcome measure in therapeutic trials and its relationship to symptom onset

**DOI:** 10.3109/21678421.2016.1140786

**Published:** 2016-02-11

**Authors:** Malcolm Proudfoot, Ashley Jones, Kevin Talbot, Ammar Al-Chalabi, Martin R. Turner

**Affiliations:** ^a^ Nuffield Department of Clinical Neuroscience, University of Oxford; ^b^ Department of Clinical Neuroscience, Institute of Psychiatry, King’s College London, LondonSE5 8AF, UK

**Keywords:** Motor neuron disease, survival, prognosis, modelling, clinical trial

## Abstract

The reduction in ALS Functional Rating Score (ALSFRS) from reported symptom onset to diagnosis is used to estimate rate of disease progression. ALSFRS decline may be non-linear or distorted by drop-outs in therapeutic trials, reducing the reliability of change in slope as an outcome measure. The PRO-ACT database uniquely allows such measures to be explored using historical data from negative therapeutic trials. The decline of functional scores was analysed in 18 pooled trials, comparing rates of decline based on symptom onset with rates calculated between interval assessments. Strategies to mitigate the effects of trial drop-out were considered. Results showed that progression rate calculated by symptom onset underestimated the subsequent rate of disability accumulation, although it predicted survival more accurately than four-month interval estimates of δALSFRS or δFVC. Individual ALSFRS and FVC progression within a typical trial duration were linear. No simple solution to correct for trial drop-out was identified, but imputation using δALSFRS appeared least disruptive. In conclusion, there is a trade-off between the drive to recruit trial participants soon after symptom onset, and reduced reliability of the ALSFRS-derived progression rate at enrolment. The need for objective markers of disease activity as an alternative to survival-based end-points is clear and pressing.

## Introduction

ALS is a heterogeneous condition with multiple pathological pathways culminating in overlapping disease phenotypes (1). Despite this complexity, clinical and demographic characteristics readily assessed at time of presentation can inform prediction of disease progression (2–7). However, even within clinically-defined subtypes (8) or genotypes (9,10), progression rates remain relatively dispersed. Accurate characterization of multimodal biomarkers that can capture the range of the pathogenic process in ALS remains a priority (11).

The successful application of future therapies for ALS depends upon meaningful evaluation against valid and relevant outcome measures. Clinical severity scores, whether measured by self-reported loss of function scales (most commonly the revised ALS Functional Rating Score, ALSFRS-R), or by objective structured assessment (e.g. the Appel Scale), have been reported to decline in a largely linear fashion (12–14). Not all studies concur, in particular early stages of the illness have been characterized by accelerated functional decline (15), although this effect is at least partially mitigated by restricting analysis to a survivor subgroup (7). The measured rate of decline remains an important predictor of functional and mortality outcomes (16,17). Change in ALSFRS over time (δALSFRS) (Figure 7 in (18)) can serve as an approximate measure of disease severity and cohort studies have demonstrated δALSFRS to predict survival (19), encouraging its inclusion as a covariate in the analysis of interventional studies. Irrespective of function, ventilatory failure is the typical mode of death in ALS, and forced vital capacity (FVC) measurement complements the ALSFRS in both prediction of survival and representation of disease progression (20–23).

The self-reported date of symptom onset is a critical framing heuristic in ALS that is taken as time-zero for calculation of δALSFRS at the first clinical encounter. This estimation is subject to recall bias, and typically relies on perception of first actual weakness, which can (albeit in a minority) neglect onset in cognitive or respiratory domains and more non-specific symptoms such as cramp or fasciculation. δFVC may similarly be estimated at the first clinical encounter by considering time-dependent deviation from predicted FVC based upon age, height and gender (13,24).

Repeated assessment of patients is inherent to most clinical trials in ALS, and permits repeated calculation of δALSFRS. Longitudinal analysis of disability progression also serves to expose selective withdrawal from trials of the most disabled participants. This potential confound is a complicating factor in the interpretation of clinical trial outcomes but prospective δALSFRS extrapolation may assist to mitigate drop-out bias. Using pooled data from therapeutic trials in ALS, we sought to compare subjectively-reported symptom onset with that calculated from repeated-interval derivations of δALSFRS extrapolated backwards.

## Methods

Demographic and clinical data from 17 therapeutic trials comprising 4752 records in PRO-ACT were included in the analysis on the basis of at least two time-separated assessments of disability (25). The majority of PRO-ACT records contain only original ALSFRS, rather than revised (ALSFRS-R) scores. Therefore, for harmonization, 882 records were converted from ALSFRS-R to ALSFRS by collapsing across the respiratory subscore (discounting orthopnoea). A total of 42,584 assessments across a mean individual maximum time-span of 11.1 (5.4) months were analysed, averaging 8.8 (3.6) assessments per person.

Time elapsed from symptom onset was recorded for over 99% of the included participants, thus defining reported disease duration. The δALSFRS (points decline per month) could therefore be calculated as either drop in ALSFRS from 40 divided by disease duration, or by subtraction of time-separated ALSFRS assessments divided by the inter-visit time-interval. Calculated δALSFRS was then used to extrapolate back to the date at which ALSFRS = 40, i.e. no disability.

This approach was then validated using FVC data and ALSFRS-R scores. 4168 records in PRO-ACT include at least two FVC measurements separated by at least one month. The smaller portion of PRO-ACT records with ALSFRS-R was supplemented by 217 individual longitudinal data records from the Lithium Carbonate in ALS (LiCALS) study, resulting in 1709 individual records suitable for analysis, that demonstrated incremental disability over a median time-interval of 11.0 months.

Within the PRO-ACT database 1863 individual records included mortality data, and from this subset only 464 remained alive at the last census. Hazard curves were constructed from both the entire population and the mortality subset to represent time-dependent risk of death or significant disability (defined as ALSFRS < = 21, the median final assessment ALSFRS across participants).

Trial drop-out mitigation methods appraised included the re-assignment of missing values with either (1) imputed values of ALSFRS based on linear extrapolation using δALSFRS calculated from the first assessment to the last available assessment, or (2) ALSFRS values carried forward unchanged from the last available assessment for that participant, with or without (3) assignment of ALSFRS = 0 if the participant died prior to the planned assessment.

Data were analysed using Matlab and SPSS 21. Paired *t*-tests compared alternative measures for individual patients and Spearman’s rho was used for correlations to minimize the effect of non-normative data. Mean value was followed by standard deviation in parenthesis.

## Results

**Figure 1.**
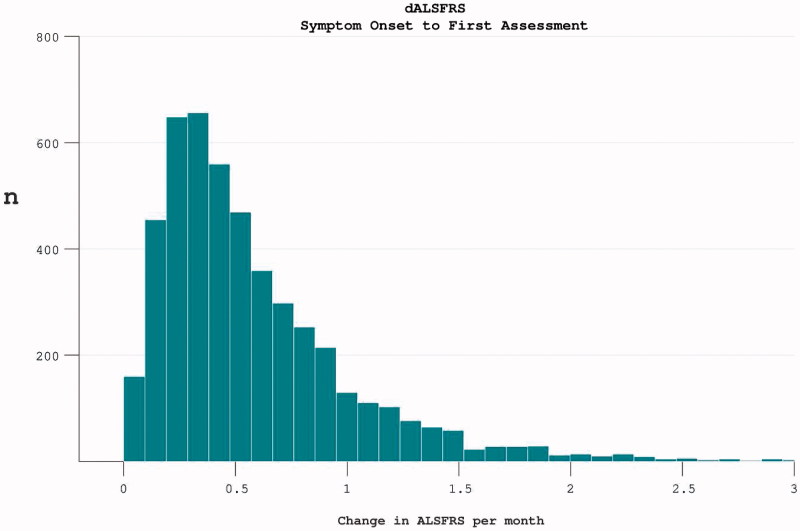
The rate of disease progression in participants at the time of trial enrolment. Calculated as (40-enrolment ALSFRS)/months from symptom onset.

At trial enrolment, the median length of disease duration was 18.7 months (mean 22.5 (14.5)). The mean rate of disease progression since symptom onset was 0.59 (0.49) per month. A wide spread of progression rate was noted, with positive skew (Figure 1).

**Figure 2.**
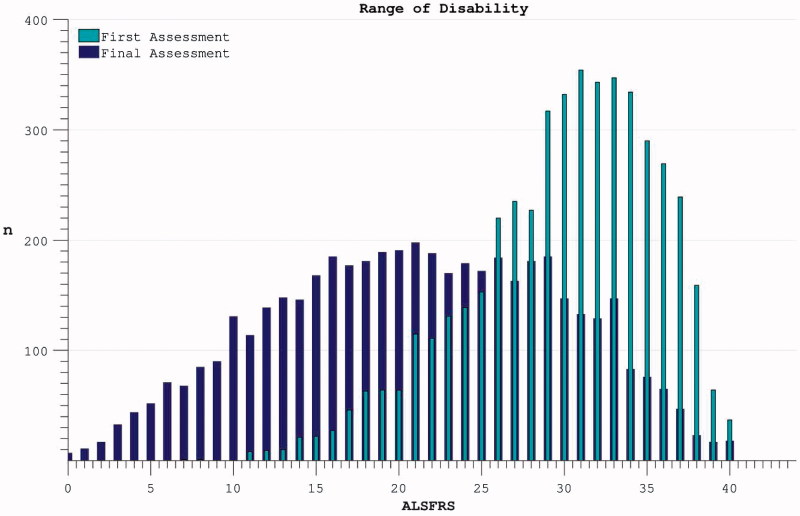
The spread of disability levels as measured by ALSFRS at both the time of trial enrolment and at the last available time-point (trial termination) for each individual.

Averaging across participants the ALSFRS dropped over each individual’s maximum-recorded interval during the trial from 29.9 (5.7) to 21.2 (8.6) (Figure 2). This variability reflected both inter-individual diversity in progression rates and inter-trial differences in length of follow-up.

**Figure 3.**
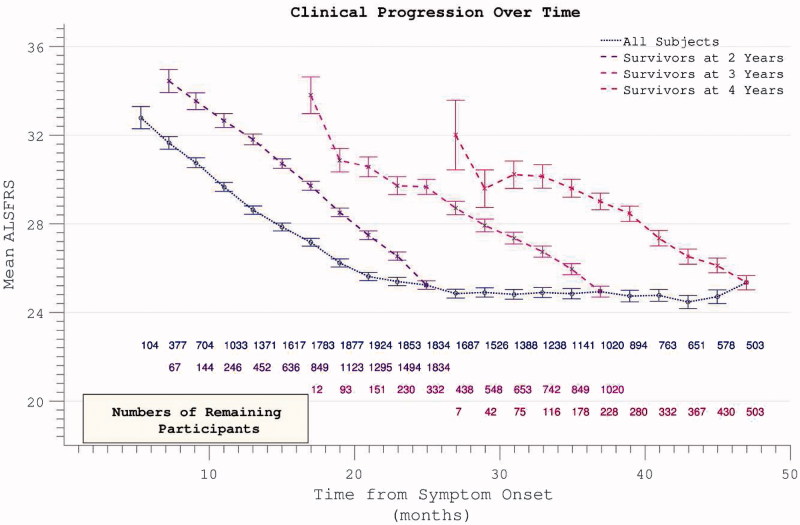
Accumulation of disability over time appears to slow over the course of the trial (dotted line), but declining numbers of participants are noted over time. Progression within groups of survivors to defined end-points appears more linear.

**Figure 4.**
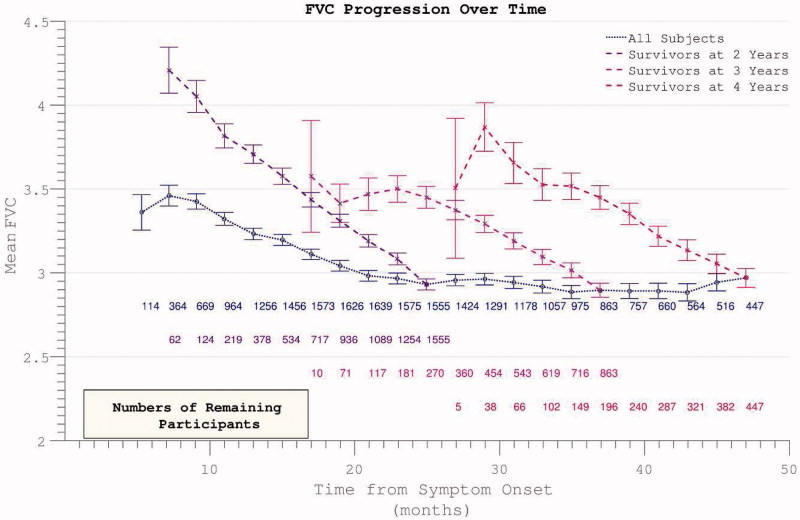
Decline in FVC mirrors ALSFRS in demonstrating apparent slowing across all participants but linear decline within survivor sub-groups.

Longitudinal analysis was complicated by declining participation over time. Patients with less rapidly progressive disease were increasingly over-represented as follow-up extended. Global PRO-ACT ALSFRS averages superficially suggested a deceleration in the rate of disability accumulation in the later stages of disease durations. However, analysis of progression curves divided into sub-groups of survivors at pre-defined yearly intervals, revealed a linear progression (linear fit *r* ^2^ = 0.998, two-year survivor group) in disability over the study period (Figure 3). Analysis of the FVC data confirmed linear decline (*r* ^2^ = 0.992) in respiratory function over time within survivor sub-groups (Figure 4).

**Figure 5.**
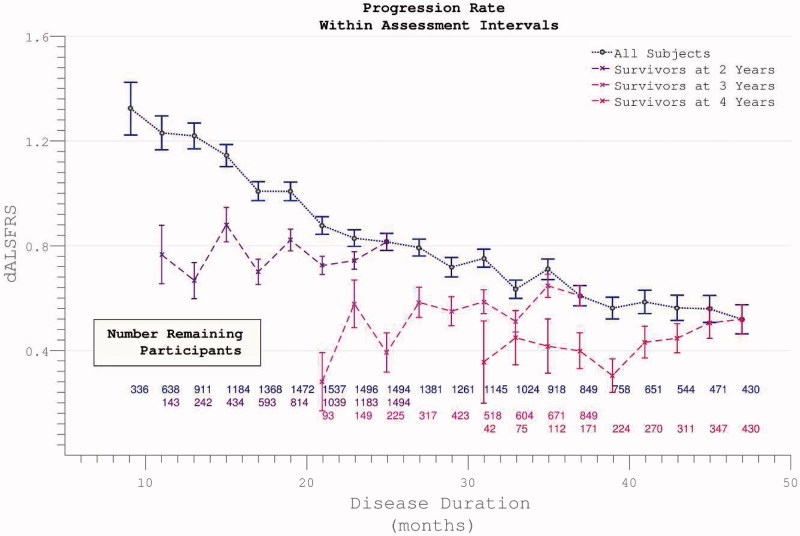
The rate of disease progression during the trial (calculated between assessment intervals) does not alter if analysis restricted to survivors. Including all participants (dotted line), the rate of disease progression appears to fall as more rapidly progressive patients drop out.

The apparent dramatic slowing in disability accumulation in the later stages of ALS appeared not to be a confounding feature specific to the ALSFRS scale, but rather to be mediated by selective loss to the trial through death or withdrawal of those with more rapidly progressive disease. This interpretation was reinforced by inspection of rates of disability accumulation as calculated across intervals between trial assessments. These rates remained constant within subgroups of survivors to specified end-points (Figure 5).

**Figure 6.**
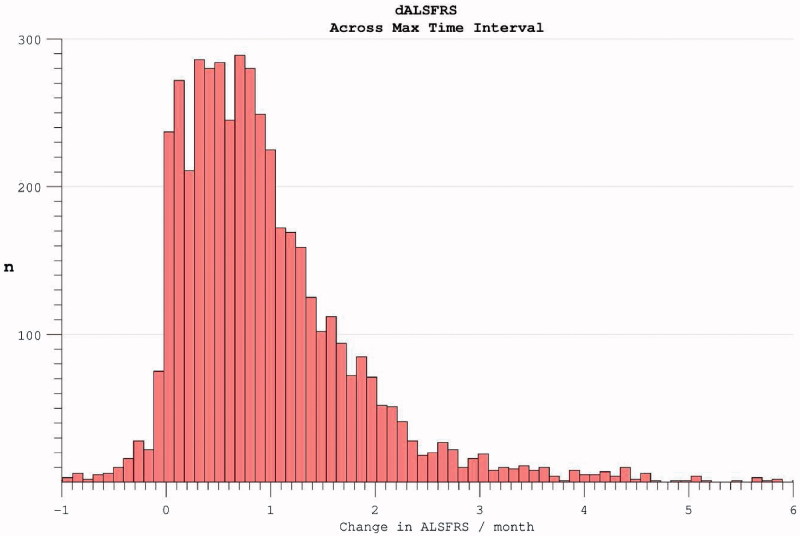
The rate of disability accumulation over the course of the trial, ignoring the time-period from symptom onset to trial enrolment.

The rate of disease progression as measured across each given individual’s maximum assessment interval was also broad with positive skew, mean 0.95 (0.96) (Figure 6).

**Figure 7.**
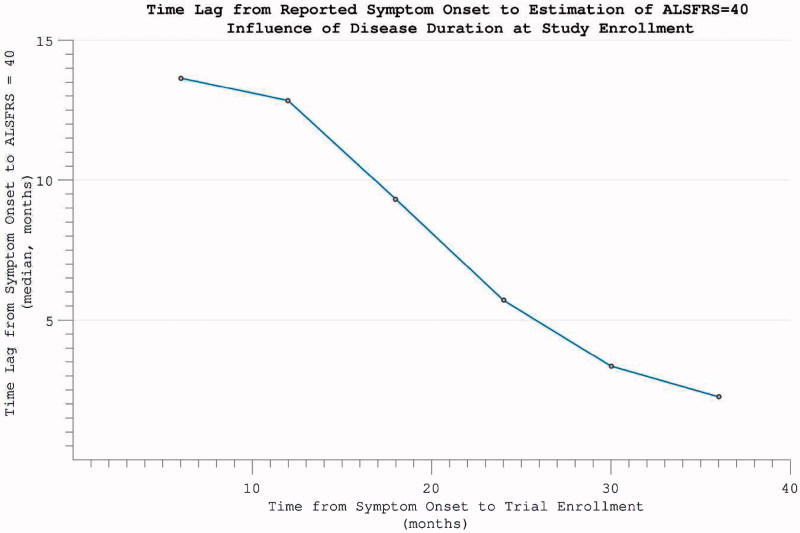
The time-difference (median = 5.8 months) between reported symptom onset and our estimate of when ALSFRS = 40 (based on individual ALSFRS gradient during trial). Later enrolment allows more accurate prognostication for an individual, as more time has passed for disability accumulation.

Considering the impact of including patient-reported symptom onset data in the calculation of disease progression rates, direct comparison was possible in 4649 individuals for whom symptom onset dates were available. This revealed the maximum interval ALSFRS measurement-derived rate to be faster (median difference 0.24 month^−1^, mean 0.37 (0.93)) than that estimated by drop in ALSFRS from symptom onset to trial enrolment (*p* < 0.001, *t*(4648) = 27.10). A correlation was nonetheless still noted between the rates as calculated by differing methods (rho = 0.421, *p* < 0.001).

**Figure 8.**
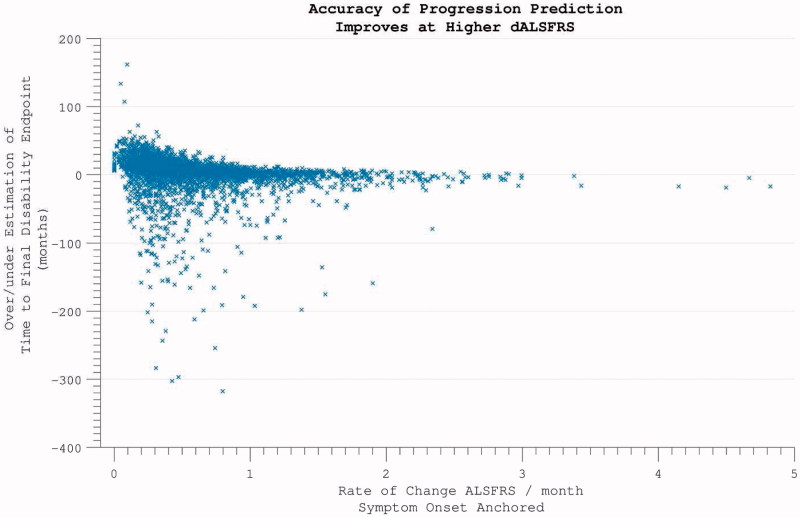
Predicting disease time-course from symptom onset. Within individuals with more rapidly progressive disease, using the symptom onset to enrolment rate to predict disease duration was highly accurate. For individuals with slower disease progression, greater inaccuracy is introduced.

Excluding 354 individuals who did not accumulate any disability over the course of study participation, the time-point of ALSFRS = 40 was estimated by extrapolating backwards from the final assessment, using the rate of progression calculated across the maximum assessment interval, under the assumption of linearity. This estimated time-point did not coincide precisely with reported symptom onset *(p*  < 0.001). Functional disability severe enough to register on the ALSFRS followed symptom onset by a median lag of 5.8 months. This interval between 1) the time-point that a patient first perceived a motor abnormality, and 2) the temporal estimation of when functional disability accumulation was first detectable on the ALSFRS scale, was found to be smaller the further into disease duration that trial enrolment occurred (Figure 7). This predictive accuracy was, however, also dependent upon the individual’s rate of disease progression, with the largest inaccuracy for those with slowest disease progression *(*‘funnel’ appearance to scatterplot Figure 8).

The validation data analysis using the ALSFRS-R described a decline from a median of 39 at trial entry to 29 at final assessment. Disability accumulation curves over time demonstrated a similar flattening of progression rates, dependent on selective loss to follow-up (Supplementary Figure 1). The time-point of first significant disability milestone (ALSFRS-R = 48) was again estimated by back-extrapolation from the final assessment point using maximum interval δALSFRS-R. The median time-lag from reported symptom onset was 8.3 months. Disability accumulation, regardless of scale choice, was subject to wide variability between individuals (Supplementary Figure 2).

**Figure 9.**
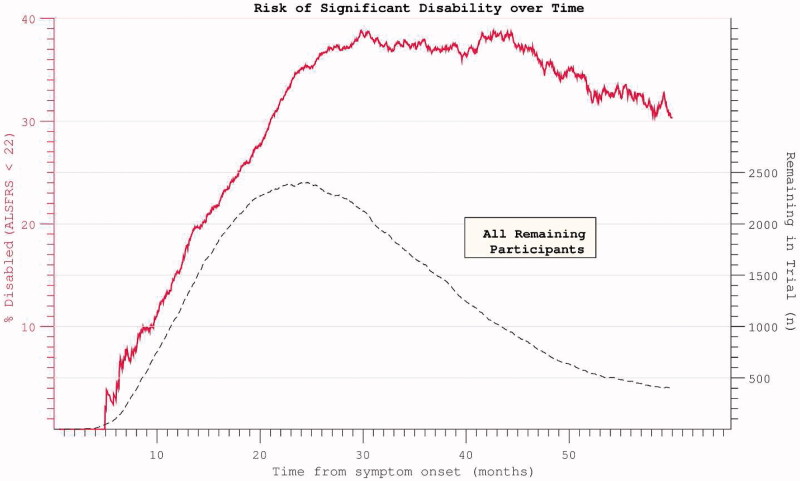
Hazard ratio of progression to significant disability (ALSFRS < = 21, the median final ALSFRS recorded) appears to plateau and diminish with time. Complicated by declining trial participation.

**Figure 10.**
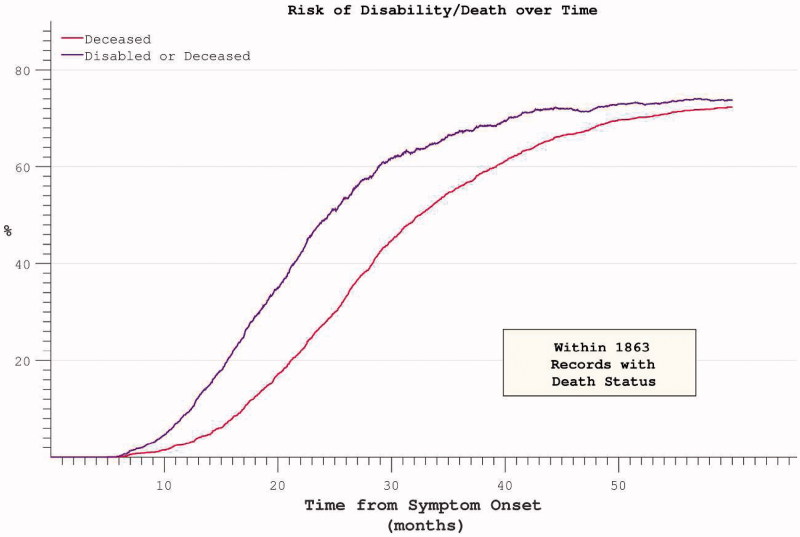
Hazard ratios for both disability and death appear to plateau despite restricting analysis to participants with mortality data at census.

**Figure 11.**
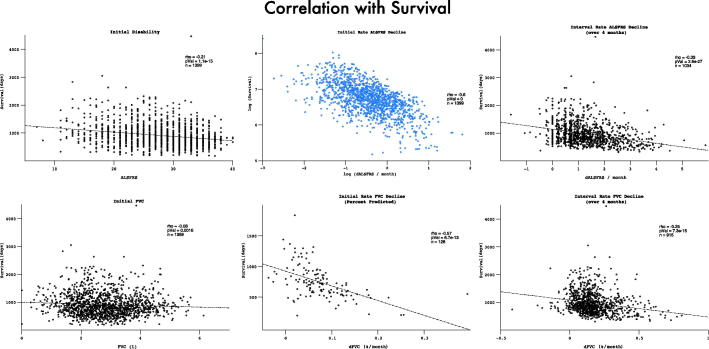
FVC and ALSFRS correlations with mortality, measured as initial absolute values, initial rates and interval rates. Initial rates predict mortality most accurately.

The median survival within the PRO-ACT mortality subset was 32.4 months from symptom onset, while median survival without significant disability was 24.4 months. Ongoing active participation in the clinical trials was noted to decline rapidly beyond 25 months after trial onset. In accordance with the above findings, the risk of acquiring significant disability appeared to plateau and then decline (Figure 9). Restricting the hazard ratio analysis to the survivor subset continued to demonstrate a plateau in mortality risk at approximately 50 months after symptom onset. Mortality was typically preceded by significant disability (Figure 10). Both FVC and ALSFRS at the initial visit were predictive of subsequent mortality, but symptom onset-anchored initial rates of decline improved predictive accuracy (strongest correlation with survival was initial δALSFRS, rho = 0.6, log-log transform displayed), and still outperformed the interval δALSFRS and δFVC derived over the first four months of trial participation (Figure 11).

**Figure 12.**
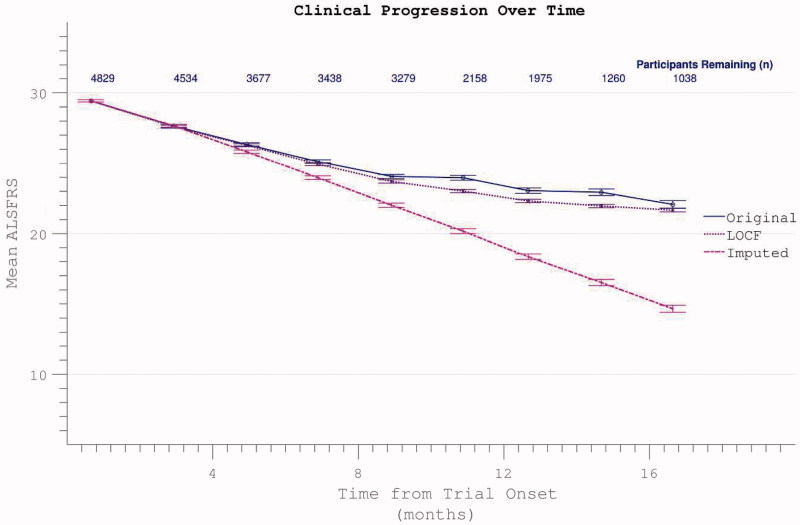
Possible dropout mitigation strategies include last observation carried forward (LOCF) or imputation of the missing values using prior δALSFRS. Imputation maintains linear progression of disability accumulation.

**Figure 13.**
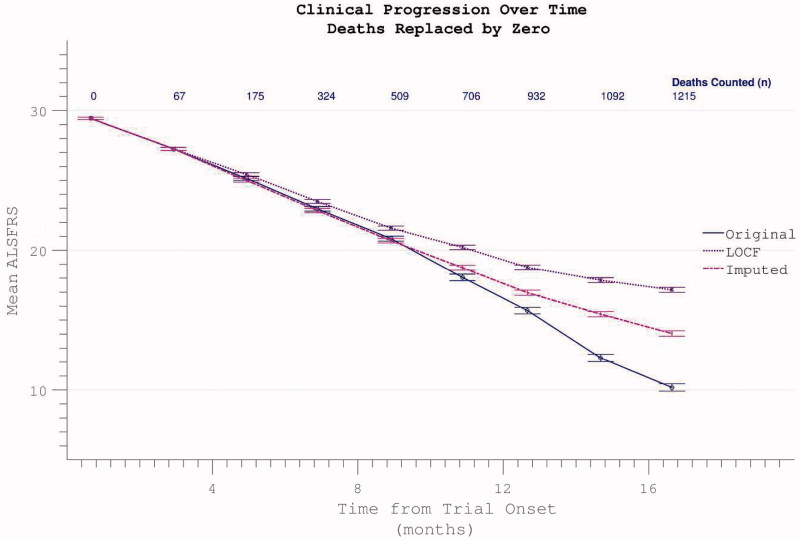
Confirmed participant death can be acknowledged by assigning zero values to subsequent ALSFRS scores, resulting in spurious apparent acceleration of disability progression. This effect is mitigated if other missing data (due to non-death trial withdrawal) are imputed by slope or carried forward (LOCF).

Comparative appraisal of drop-out mitigation options revealed the last-observation-carried-forward (LOCF) technique to only minimally correct the apparent flattening in the δALSFRS slope, whereas a more linear progression was revealed using slope-imputed values (Figure 12). Assigning ALSFRS = 0 to assessments falling after the confirmed date of a participant’s death resulted in a disproportionate acceleration of apparent ALSFRS decline unless the remaining absent values were re-assigned by imputation or LOCF (Figure 13).

## Discussion

The key findings of this study were:

- Initial rates of ALS progression calculated from recalled date of symptom onset tended to underestimate the subsequent rate of disability progression.
- The recalled symptom onset-anchored rate of progression nevertheless predicted survival more accurately than four month interval estimates of δALSFRS or δFVC.
- As the denominator of time was increased, symptom onset-anchored progression rate improved in its predictive power of future progression rate.
- After comparatively slower initial progression from symptom onset, individual ALSFRS and FVC progression during a typical trial duration appeared to be linear.
- No simple solution to correcting for trial drop-out was identified, but imputation using δALSFRS appeared least disruptive.

These findings are relevant for future studies that intend to take account of δALSFRS in participant enrolment, consideration of covariables, or end-point selection. These data support the application of the longest possible time-intervals as denominator in calculation of δALSFRS to minimise chance variation. These data also support previous findings that the rate of disease progression calculated from symptom onset carries valuable prognostic information (13,16,18,19).

Significant discrepancy exists in the rate of disease progression when calculated with or without the initial time-point of reported symptom onset (26). It seems probable that symptoms of ALS may be noticeable by a patient (or family members, who often contribute to the estimation of disease onset in our experience) without causing any significant disability. Therefore the ALSFRS might lack sensitivity at the milder end of the disease spectrum. Conversely, it may be that significant disability milestones are rapidly attained in the middle stages of ALS (i.e. around the time of enrolment in the trial) without any acceleration of the disease process itself. In the last stages of advanced disability, the ALSFRS might well ‘bottom out’ (27), although most clinical trials do not include these data; thus the imputation choice of zero to represent death remains necessarily arbitrary. FVC data correspondingly appeared to change little in last months before death (23) and were particularly prone to inaccuracy in the context of significant bulbar weakness (28).

Progression and survival in ALS have been modelled using a Weibull probability distribution (17,29), but since longitudinally extensive data demonstrate a plateau effect in mortality risk (Figure 8), previous investigators have resorted to a compound distribution (30), suggesting this model to have limited application. Untransformed data clearly demonstrate prolonged survival in a minority of patients (Figure 9).

Systematic reviews of prognostic factors in ALS have been performed with respect to both survival (31) and functional status (32). These factors overlap but are not identical; however, reliable models for prediction of function are limited by a relative paucity of replicated studies (6,7). The PRO-ACT database has already contributed greatly, for example, in confirming that higher baseline levels of serum urate and creatinine are independently associated with slower ALSFSR-R decline and longer survival (25), and in the formulation of a multivariate predictive model using Bayesian trees (33). Historical accounts of long-term survival in ALS noted a plateau in cumulative mortality around five years from disease onset (2,30), and these findings continue to be replicated in the modern era (4,34–36). Mortality remains the gold standard outcome measure for future ALS clinical trials, but inclusion of novel measures of function has been proposed (37) with the goal of addressing some of the documented inadequacies of the ALSFRS-R (38) and perhaps simultaneously accounting for the confounding impact of mortality (29).

The ALSFRS has been reported to misjudge disease severity when patients underestimate muscle function in contrast to objective examination (39). The relatively small time-span between assessments in clinical trials is also perhaps more prone to random error. It is also possible that cognitive impairment might contribute to an inaccurate recall of time-scale for symptom onset. The cognitive profile of ALS is typified by behavioural, executive and language dysfunction (40), but impairment of memory is also reported (41), and qualitatively distinct to that found in amnestic mild cognitive impairment (42). Inaccuracy of recall would adversely affect the prognostic value of symptom onset calculations but should not result in such systematically biased estimation of symptom duration.

An important future consideration would be whether rates of progression measured by alternative tools might be less susceptible to this symptom onset bias, thus potentially clarifying the bias inherent to the ALSFRS scale itself. Such analyses should include change in examination-based scales such as staging (43) and neurophysiological indices such as motor unit number estimation (MUNE), shown to be sensitive to more slowly-progressive disease (44). Alternative techniques to measure respiratory function in ALS have been assessed to outperform FVC in both sensitivity to detect deficits (45,46) and prognostication (47). They may, however, share some limitations (48,49), particularly since different respiratory abnormalities do not evolve simultaneously and also partially respond to therapeutic intervention (50).

This analysis meanwhile serves to remind of the potential for measurement techniques to bias the outcome of even the best-designed prospective interventional studies and reinforces the need for simultaneous assessment of more than one functional outcome measure (20), corroborated by multi-modal biomarker development (51).

## Supplementary Material

suppFigure2.eps

suppFigure1.eps

## Notes

**Supplementary material available online** Supplementary Figures 1 and 2
